# Correlation between Viral Wastewater Concentration and Respiratory Tests, Oregon, USA

**DOI:** 10.3201/eid3010.240637

**Published:** 2024-10

**Authors:** Noah Lininger, Rebecca Falender, Paul Cieslak, Arilene Novak, M. Andraya Hendrick, Devrim Kaya, Casey Kanalos, Oumaima Hachimi, David Mickle, Christine Kelly, Tyler Radniecki, Melissa Sutton

**Affiliations:** Oregon Health Authority, Portland, Oregon (N. Lininger, R. Falender, P. Cieslak, A. Novak, M.A. Hendrick, M. Sutton);; Oregon State University, Corvallis, Oregon, USA (D. Kaya, C. Kanalos, O. Hachimi, D. Mickle, C. Kelly, T. Radniecki).

**Keywords:** COVID-19, wastewater surveillance, wastewater-based epidemiology, environmental surveillance, SARS-CoV-2, coronavirus, influenza, respiratory syncytial virus, correlation study, viruses, Oregon

## Abstract

We evaluated the association between wastewater concentration and weekly percent positivity of patient testing for SARS-CoV-2, influenza, and respiratory syncytial virus in Oregon, USA. We found strong, positive correlations for SARS-CoV-2 (ρ = 0.84, p<0.001), influenza (ρ = 0.73, p<0.001) and respiratory syncytial virus (ρ = 0.69, p<0.001).

Since the 1940s, wastewater surveillance has been used to track pathogens that are shed in feces (*1*). Testing wastewater for respiratory viral pathogens avoids potential biases found in case-based surveillance methods. Wastewater surveillance captures information from persons who are asymptomatic or not ill enough to seek care, who reside in rural areas or underserved communities with limited access to care, or who test at home and do not report their results (*2*).

Community transmission of SARS-CoV-2, influenza, and respiratory syncytial virus (RSV) are primarily monitored through test percent positivity. Previous studies have found positive regional correlations between wastewater viral concentration and percent positivity for COVID-19, influenza, and RSV (*3*–*5*). In this study, we compared weekly SARS-CoV-2, influenza, and RSV wastewater concentrations with patient test positivity during September 6, 2020–May 11, 2023, in Oregon, USA.

Our study included wastewater-treatment facilities that submitted >1 sample to Oregon’s surveillance system during September 6, 2020–May 11, 2023. Sites located on sovereign tribal territories were excluded. Wastewater testing for SARS-CoV-2 was performed year-round. Influenza and RSV wastewater testing was conducted during the influenza and RSV respiratory seasons (September 15, 2021–June 24, 2022, and August 28, 2022–April 30, 2023, for influenza; August 22, 2022–April 30, 2023, for RSV). We collected 24-hour composite samples from wastewater treatment facility influents 1–2 times weekly. We quantified SARS-CoV-2, influenza, and RSV RNA concentrations by using reverse transcription droplet digital PCR, as described previously (*6*). We derived primers and probes for SARS-CoV-2 testing from the 2019-nCoV CDC droplet digital PCR triplex probe assay (BioRad Laboratories, https://www.bio-rad.com) and those for influenza testing from the Center for Disease Control and Prevision influenza SARS-CoV-2 multiplex assay (Integrated DNA Technology, https://idtdna.com); we adopted those for RSV testing from previous studies (*7*). We calculated statewide weekly wastewater viral concentrations by averaging log gene copies per person per day normalized for population and flow across all participating sites. For sites that submitted >1 sample per week, the average concentration of the 2 samples was used.

Human test positivity is the count of positive patient tests divided by the count of tests performed. The National Respiratory and Enteric Virus Surveillance System (NREVSS) is a sentinel laboratory surveillance system that collects aggregate patient test results for 7 viral pathogens including SARS-CoV-2, influenza, and RSV (*8*). A total of 24 sentinel laboratories in Oregon are registered in NREVSS. 

We used pairwise correlation to assess the relationship between the statewide weekly wastewater concentration and human test positivity for each pathogen. We assessed the normality of continuous variables with a visual inspection of histograms and quantile-quantile plots and the Shapiro-Wilk test (α = 0.05). The assumption of normality was not met, so we used the Spearman rank correlation (ρ) for all analyses. We used SAS 9.4 (SAS Institute Inc., https://www.sas.com) for the analyses.

A total of 48 wastewater treatment plants, serving 62.3% of the population of Oregon, submitted >1 sample during the study period. Of 7,185 wastewater samples tested for SARS-CoV-2, a total of 6,910 (96.2%) were positive; of 4,081 wastewater samples tested for influenza, 767 (18.8%) tested positive; and of 1,689 wastewater samples tested for RSV, 473 (28.0%) tested positive. We paired human test positivity with statewide viral concentrations, by week, over 140 weeks for SARS-CoV-2, over 76 weeks for influenza, and over 36 weeks for RSV. We found strong positive correlations between wastewater concentration and human test positivity for SARS-CoV-2 (ρ = 0.84, p<0.0001), influenza (0.73, p<0.0001), and RSV (ρ = 0.69, p<0.0001) (Figure).

**Figure:**
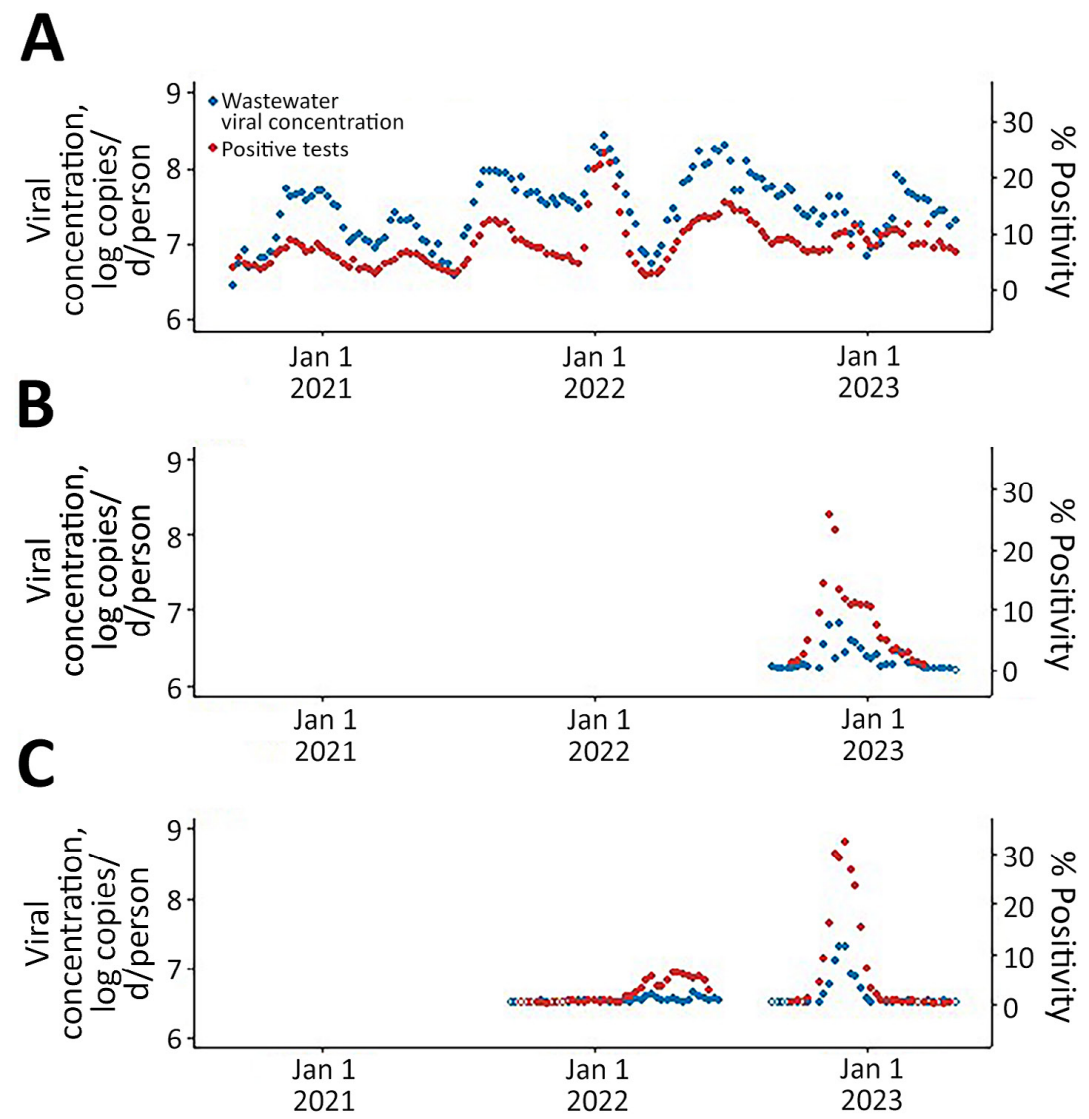
Statewide weekly wastewater viral concentration (log copies/d/person) and clinical human test positivity over time for respiratory pathogens, Oregon, USA, September 6, 2020–May 11, 2023. A) SARS-CoV-2; B) respiratory syncytial virus; C) influenza. Nondetect values were assigned the mean 1/2 limit of detection for each pathogen. Open circles indicate no detections for that week.

A limitation of this study is that wastewater surveillance excludes people without access to municipal sewer service. Septic tanks are more prevalent in rural communities than in urban communities, which might have biased our results if people outside of sewersheds are infected at different rates than those within. In addition, whereas SARS-CoV-2 remained a reportable disease throughout the study period, influenza and RSV did not. Fewer influenza and RSV cases might have been reported (outside of NREVSS) during the study period, and statewide human test positivity metrics might not be fully representative of the 3 pathogens, weakening their associations with statewide wastewater data.

Our study found positive correlations between wastewater viral concentration and human test percent positivity for SARS-CoV-2, influenza, and RSV. The strength of association observed suggests wastewater surveillance acts as an indicator of community transmission for those pathogens. Wastewater data are not affected by healthcare-seeking behavior or testing biases, can be analyzed nearly in real-time from pooled community samples, and can be localized to the sewershed level, informing local public health decisions. Our results demonstrate how wastewater surveillance can strengthen our understanding of SARS-CoV-2, influenza, and RSV community transmission.
